# Protocol for the efficacy and safety of fecal microbiota transplantation in children with autism spectrum disorder: a prospective single-center, single-arm interventional study

**DOI:** 10.3389/fped.2025.1660773

**Published:** 2025-12-19

**Authors:** Baixian Lin, Zhongsheng Zhu, Xiao Yang, Ziyuan Li, Haokui Zhou, Mingjing Luo, Jiaojie Guan, Yigui Zou, Hu Chen, Zeling Zhuang, Shiyun Meng, Wenwen Li, Qinghua Yang, Dongling Dai

**Affiliations:** 1 International Medical Center, Endoscopy Center and Gastroenterology Department, Shenzhen Children’s Hospital, Shantou University Medical College, Shenzhen, China; 2 State Key Laboratory of Quantitative Synthetic Biology, Shenzhen Institute of Synthetic Biology, Shenzhen Institute of Advanced Technology, Chinese Academy of Sciences, Shenzhen, China; 3 Department of Biostatistics, School of Public Health, Cheeloo College of Medicine, Shandong University, Jinan, China; 4 Shenzhen Children’s Hospital, Southern University of Science and Technology, Shenzhen, China; 5 Shenzhen Children’s Hospital, The Chinese University of Hong Kong, Shenzhen, China; 6 Shenzhen Children’s Hospital, Shenzhen University Health Science Center, Shenzhen, China

**Keywords:** autism spectrum disorder, fecal microbiota transplantation, microbiota-gut-brain axis, microbiome, gastrointestinal symptoms, microbiota dysbiosis, clinical protocol

## Abstract

**Background:**

Autism spectrum disorder (ASD) is a neurodevelopmental condition affecting 0.7% of children globally, with 90% experiencing comorbid gastrointestinal (GI) symptoms. Fecal microbiota transplantation (FMT) may modulate ASD symptoms via the microbiota-gut-brain axis (MGBA).

**Methods:**

This open-label single-arm trial enrolls 30 children (2–12 years) with moderate-to-severe ASD, defined as a Childhood Autism Rating Scale (CARS) score of ≥36. Participants receive 3 nasojejunal FMTs (5 mL/kg) over 5 days. The primary outcomes are GI symptom improvement, assessed using the Gastrointestinal Symptom Rating Scale (GSRS), and ASD severity, assessed using the CARS. Secondary outcomes include social responsiveness (Social Responsiveness Scale, SRS), aberrant behaviors (Aberrant Behavior Checklist, ABC), and gut microbiota changes assessed by metagenomic next-generation sequencing (mNGS).

**Ethics and dissemination:**

Ethical approval obtained from Shenzhen Children's Hospital Ethics Committee. Results will be disseminated via peer-reviewed publications and conference presentations.

**Clinical Trial Registration**: https://www.chictr.org.cn/showproj.html?proj=229136, identifier ChiCTR2400083998. Registered on 2024-05-08. Registered title: “Efficacy and safety of fecal microbiota transplantation in treatment of autism spectrum disorder: a prospective single-center intervention study”.

## 1 Introduction

Autism Spectrum Disorder (ASD) is a complex neurodevelopmental disorder characterized by core features of social communication deficits, restricted interests, and repetitive behaviors, often accompanied by metabolic dysregulation, immune dysfunction, and gastrointestinal (GI) symptoms (1). The global prevalence of ASD is approximately 0.6%, with a significant upward trend, making it a critical public health concern in pediatric mental health (2).While the exact etiology of ASD remains incompletely understood, the interplay between genetic and environmental factors is recognized as the primary pathogenic mechanism (1, 3). In recent years, gut microbiota dysbiosis and its impact on neurodevelopment via the “Microbiota-Gut-Brain Axis” (MGBA) have emerged as a research focus (4).Fecal Microbiota Transplantation (FMT), an innovative therapy to modulate gut microbiota, offers a novel direction for ASD treatment (5, 6).

In addition to core behavioral manifestations, approximately 90% of children with ASD experience comorbid GI symptoms such as bloating, abdominal pain, constipation, or diarrhea, which are positively correlated with ASD severity (7). Epidemiological data indicate a marked increase in ASD prevalence over the past decade. For instance, the prevalence among 8-year-old children in the United States rose from 1.1% in 2008 to 2.3% in 2018 (8). In China, the first multicenter, large-scale epidemiological study reported a prevalence of 0.7% in children aged 6–12 years, with a male-to-female ratio of approximately 4:1 (9). Although genetic factors dominate ASD pathogenesis (1), environmental contributors such as perinatal infections and antibiotic exposure also play critical synergistic roles (10). Current clinical management of ASD primarily relies on behavioral interventions (e.g., Applied Behavior Analysis) and pharmacotherapy (e.g., risperidone, aripiprazole). However, the efficacy of behavioral therapies requires further validation (11), while medications are limited by adverse effects such as extrapyramidal symptoms and metabolic disturbances (12). Consequently, the exploration of safe and effective therapeutic strategies remains urgent.

The gut microbiota, also termed gut microbiota, constitutes a complex ecosystem comprising bacteria, viruses, fungi, and protozoa, predominantly residing in the colon. Bacteria account for over 99% of this ecosystem, with approximately 40 trillion bacterial cells and more than 1,000 species in the human gut. The diversity and functional homeostasis of gut microbiota are critical for host metabolism, immunity, and neurodevelopment (13, 14). In healthy individuals, Firmicutes and Bacteroidetes phyla dominate the gut microbiota (>90% combined abundance). In contrast, ASD patients exhibit significant dysbiosis characterized by reduced *α*-diversity, increased abundance of *Bacteroides* and *Clostridioides*, and marked depletion of *Prevotella,* *Bifidobacterium*, and *Veillonella*. These alterations are closely associated with intestinal barrier disruption (“leaky gut”), systemic inflammation, and metabolic abnormalities (15, 16). Studies have revealed decreased levels of short-chain fatty acids (SCFAs) and elevated neurotoxic metabolites (e.g., isopropanol, p-cresol) in the feces of ASD children. Such imbalances may disrupt neurotransmitter equilibrium (e.g., serotonin, γ-aminobutyric acid) by crossing the blood-brain barrier, contributing to neuropsychiatric symptoms (17). Animal experiments further validate the causal role of gut microbiota: fecal transplants from ASD children into germ-free mice induce social deficits and repetitive behaviors, demonstrating microbiota-mediated modulation of the tryptophan-serotonin metabolic axis and central nervous function (18).

The gut microbiota interacts bidirectionally with the central nervous system through four pathways—neural, immune, endocrine, and metabolic—collectively forming the MGBA. Below are the potential mechanisms by which gut microbiota influences ASD-like behaviors via these pathways:

**Neural Pathway:** Gut microbiota produce short-chain fatty acids (SCFAs), such as butyrate, which activate vagal signaling and modulate neurotransmitters like glutamate and GABA, thereby affecting synaptic plasticity and neural transmission (19). Studies indicate reduced butyrate levels and aberrant glutamate metabolism in the gut microbiota of ASD children, correlating with impaired neurotransmission (19).Specific microbiota (e.g., *Lactobacillus*, *Bifidobacterium*) synthesize neuroactive compounds (e.g., GABA, serotonin precursors), directly or indirectly modulating the excitatory-inhibitory balance in the central nervous system. Notably, fecal GABA levels are reduced in ASD children, and probiotic supplementation has been shown to restore neurotransmitter metabolism (20).
**Immune Pathway:** Dysbiosis (e.g., increased Proteobacteria abundance) disrupts intestinal barrier integrity, leading to “leaky gut,” which allows pro-inflammatory molecules like lipopolysaccharides (LPS) to enter systemic circulation. This triggers systemic inflammation, and cytokines (e.g., IL-6, TNF-α) exacerbate neuroinflammation by crossing the blood-brain barrier, worsening ASD symptoms (21). Gut microbiota also regulate immune homeostasis by balancing Th17/Treg cell activity. Enhanced Th17 pro-inflammatory responses in ASD children may drive central neuroinflammation, with specific taxa (e.g., Clostridioides) linked to Th17 pathway activation (22).
**Endocrine Pathway:** Approximately 90% of serotonin (5-HT) is synthesized by enterochromaffin cells in the gut, relying on microbiota-dependent tryptophan metabolism. In ASD, aberrant tryptophan metabolism (e.g., enhanced kynurenine pathway) reduces serotonin synthesis, contributing to central serotonin deficiency, which impairs emotional regulation and social behavior (23). ASD children exhibit elevated colonic tryptophan hydroxylase (TPH1) expression, reduced serotonin transporter (SERT) levels, and prefrontal cortex TPH2 dysfunction (23). Additionally, gut microbiota modulates hypothalamic-pituitary-adrenal (HPA) axis activity via glucocorticoid regulation. Dysbiosis may amplify stress responses, aggravating ASD symptoms. Probiotic interventions have been shown to lower cortisol levels and alleviate anxiety in ASD children (24).
**Metabolic Pathway:** SCFAs (e.g., butyrate, propionate), produced by microbial fermentation of dietary fiber, exhibit anti-inflammatory properties, maintain gut barrier function, and regulate gene expression. Reduced butyrate levels in ASD children may exacerbate intestinal permeability and neuroinflammation (25). Studies report significantly lower fecal butyrate in ASD patients compared to controls, with symptom improvement correlating with restored SCFA levels post-intervention (25). Gut microbiota also influence neural excitability via glutamate and GABA metabolism. Dysregulated glutamate pathways (e.g., decreased 2-ketoglutarate) in ASD may disrupt prefrontal cortex function. Probiotic interventions can normalize glutamate-related metabolites, suggesting therapeutic potential (19).

In recent years, FMT has emerged as a promising alternative therapy for GI disorders. FMT involves transplanting functional microbiota from healthy donors into patients' GI tracts, restoring microbial composition and metabolism to alleviate dysbiosis and clinical symptoms (26). While FMT is guideline-recommended for recurrent *Clostridioides difficile* infection, it has also shown potential in neuropsychiatric disorders such as Parkinson's disease, depression, and ASD (27). However, it is important to acknowledge that there is a paucity of data on the use of FMT in children for indications beyond C. difficile infection, an area that requires further rigorous investigation (28). Unlike single-strain probiotics, FMT enables multidimensional modulation by transferring hundreds of microbial species, viruses, and fungi, offering comprehensive restoration of gut microbiota (26). A study demonstrated that FMT reduced Childhood Autism Rating Scale (CARS) scores by 22% and GI Symptom Rating Scale (GSRS) scores by 82%, with sustained increases in microbiota diversity and *Bifidobacterium/Prevotella* abundance for up to 8 weeks post-treatment (29).

However, FMT's clinical application remains contentious:

**Inconsistent Efficacy:** A randomized controlled trial (RCT) found no significant difference between oral FMT capsules and placebo, potentially due to donor variability or administration routes (30).
**Safety Concerns:** Although most adverse effects (AEs) are mild to moderate, risks such as infections and long-term safety in ASD patients require further evaluation (31, 32).
**Unclear Mechanisms:** While FMT alters gut microbiota composition in ASD, the pathways linking microbial changes to symptom improvement (e.g., specific metabolites or immune pathways) remain undefined, hindering biomarker-guided personalized therapy (33).
**Uncertain Long-term Outcomes:** Long-term follow-up studies report diminishing therapeutic effects over time, with partial relapse of core symptoms in some patients (31).

The complexity of ASD demands interdisciplinary collaboration. As a powerful tool to modulate the MGBA, FMT holds promise for addressing neurodevelopmental deficits at their root. Despite current limitations, advancements in mechanistic understanding, technological innovation, and clinical standardization may position FMT as a cornerstone of precision medicine for ASD, offering new hope for affected children and families. Future efforts must balance scientific rigor with clinical translation to bridge the gap between laboratory research and real-world therapeutic applications. Therefore, we hypothesize that multi-course FMT will significantly improve both GI symptoms and core behavioral deficits in children with moderate-to-severe ASD, and that these clinical improvements will be associated with the restoration of gut microbial diversity and function.

## 2 Methods and analysis

### 2.1 Study objectives

#### 2.1.1 Primary objective

To investigate the clinical efficacy and safety of FMT in children with ASD.

#### 2.1.2 Secondary objectives

2.1.2.1 To analyze changes in gut microbiota composition during FMT treatment in children with ASD.

2.1.2.2 To explore correlations between core microbiota and clinical outcomes.

### 2.2 Study design

**Figure 1:**
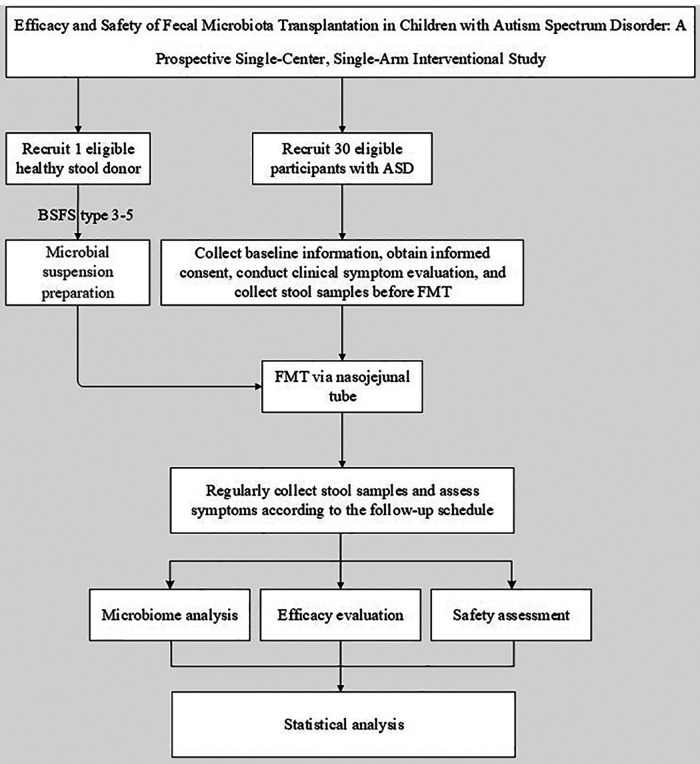
Technical route map. BSFS, bristol stool form scale; ASD, autism spectrum disorder; FMT, fecal microbiota transplantation.

This is a prospective, open-label, single-arm, single-center clinical study enrolling children aged 2–12 years with moderate-to-severe ASD. The study aims to evaluate the efficacy and safety of FMT in ASD, investigate microbial colonization patterns, and identify core microbiota post-FMT. Findings will provide evidence-based support for clinical protocols and theoretical foundations for individualized precision therapy in ASD. A technical route map for the study is shown in Figure 1. All clinical data will be recorded on standardized Case Report Forms (CRFs) and entered into a secure, password-protected electronic database to ensure data quality and integrity.

### 2.3 Study participants

#### 2.3.1 Inclusion criteria

Participants must meet all of the following criteria:

Aged 2–12 years.
Diagnosed with ASD according to the Diagnostic and Statistical Manual of Mental Disorders, Fifth Edition (DSM-5), with a CARS total score ≥36 (moderate-to-severe autism).

DSM-5 Diagnostic Criteria for ASD:

Onset in early developmental stages.
Persistent deficits in social communication and interaction across multiple contexts, accompanied by restricted, repetitive patterns of behavior, interests, or activities.
Symptoms cause clinically significant impairment in social, occupational, or other critical functional areas.
Symptoms are not better explained by intellectual disability or global developmental delay.

CARS Scoring System:

Total score <30: non-autistic.
Total score 30–36: mild-to-moderate autism.
Total score ≥36: moderate-to-severe autism.
Legal guardians fully comprehend the trial's informed consent and voluntarily provide written consent.
Compliance with follow-up visits, examinations, and specimen collection.
No probiotic supplements consumed within the preceding 3 months.
Participants on stable doses of psychopharmacological medications for at least 3 months prior to enrollment are eligible. No new psychopharmacological therapies may be initiated, and no dose changes are permitted during the study period.

#### 2.3.2 Exclusion criteria

Participants will be excluded if any of the following criteria apply:

Use of probiotics or prebiotics within 3 months prior to enrollment.
Antibiotic usage within 1 month prior to enrollment.
Presence of fever (axillary temperature ≥37.5 °C).
Dependency on tube feeding.
Severe GI conditions requiring immediate intervention (e.g., life-threatening intestinal obstruction, perforation, hemorrhage, ulcerative colitis, Crohn's disease, celiac disease, or eosinophilic esophagitis).
Diagnosis of severe malnutrition, underweight status (BMI-for-age <3rd percentile), or severe immunodeficiency disorders.
History of severe allergic reactions (e.g., anaphylaxis).
Monogenic disorders (e.g., Fragile X syndrome, Rett syndrome).
Comorbid psychiatric diagnoses, including depression, developmental speech/language disorders, intellectual disability, attention-deficit/hyperactivity disorder (ADHD), selective mutism, reactive attachment disorder, or childhood schizophrenia.

#### 2.3.3 Withdrawal criteria

Participants will be withdrawn from the study if:

The participant or legal guardian withdraws informed consent due to unwillingness or inability to continue the trial.
The participant fails to complete scheduled follow-up assessments.
Clinical deterioration renders continued participation unsafe or impractical.
Intolerable adverse reactions occur, and the investigator deems the risks of continued participation to outweigh potential benefits.

Note: Participants may withdraw from the trial at any time without justification. Investigators will document the reasons for withdrawal (if provided) in the case report form and complete all feasible assessments. Participants experiencing adverse reactions will receive appropriate medical management based on their condition.

#### 2.3.4 Elimination criteria

Participants will be eliminated from the final analysis if:

No data are recorded throughout the trial period.
Serious protocol deviations occur (e.g., unapproved concomitant therapies, non-compliance with FMT procedures).
Medications or interventions that could confound trial results are administered during the study.

### 2.4 Study procedures and content

#### 2.4.1 Recruitment, screening, and management of healthy fecal donors

Donor selection is critical to the safety of FMT. Our protocol is designed in strict accordance with international consensus guidelines, including the European Consensus Conference on Faecal Microbiota Transplantation, the International Consensus Conference on Stool Banking, and joint NASPGHAN/ESPGHAN recommendations for pediatric FMT (34, 35).

##### 2.4.1.1 Multi-step donor screening protocol

Potential donors undergo a rigorous, multi-step screening process to minimize the risk of transmitting infectious or microbiota-mediated diseases.

Step 1: Clinical Assessment and Exclusion. The initial phase involves a detailed questionnaire, a clinical interview, and a physical examination. Individuals are excluded based on:

GI Conditions: History of inflammatory bowel disease (IBD), irritable bowel syndrome (IBS), chronic constipation or diarrhea, celiac disease, or GI malignancies.
Other Medical Conditions: History of autoimmune diseases, atopic conditions (asthma, eczema), metabolic syndrome, diabetes, chronic pain syndromes, or significant neurological or psychiatric conditions.
Medication Use: Antibiotic use within the past three months; current use of immunosuppressants, systemic antineoplastics, or chronic proton pump inhibitors.
Infection Risk Factors: Recent infectious symptoms (e.g., fever), recent travel to high-risk regions, or frequent contact with healthcare settings [to mitigate risk of multidrug-resistant organism (MDRO) colonization].

Step 2: Comprehensive Laboratory Screening. Eligible candidates from Step 1 proceed to extensive laboratory testing.

Serological (Blood) Screening: Tests for HIV-1/2, Hepatitis A, B, and C, and syphilis. A complete blood count, comprehensive metabolic panel, lipid profile, hs-CRP, and fluorescent antinuclear antibody tests are also performed.
Stool Screening: Samples are meticulously tested to exclude pathogens.
Bacterial Pathogens: Salmonella, Shigella, Campylobacter, Yersinia, and *Clostridioides difficile* toxin.
Viral Pathogens: Rotavirus, adenovirus, and norovirus.
Parasites: Ova and parasites, including Giardia and Cryptosporidium.
Specialized Testing: Given the upper GI administration route, donors are screened for Helicobacter pylori (stool antigen test). In line with current safety standards, stringent screening for MDROs is conducted, including for ESBL-producing Enterobacteriaceae, VRE, CRE, and MRSA.

##### 2.4.1.2 Donor management and contingency plan

Donor Consistency: To minimize inter-donor variability as a confounding factor, all participants will receive FMT material from a single, qualified primary donor.
Contingency Plan: To prevent study interruption, a backup donor will be fully screened and qualified at the start of the trial. This backup donor will be activated only if the primary donor becomes unavailable for any reason.

##### 2.4.1.3 Ongoing donor monitoring

A qualified donor must undergo regular re-evaluation. A screening questionnaire is completed before each donation to identify any interval changes in health or lifestyle. Full serological and stool testing panels are repeated every 8–12 weeks for donors providing samples over an extended period.

#### 2.4.2 Recruitment of 30 ASD children

Thirty children aged 2–12 years with moderate-to-severe ASD (CARS score ≥36), diagnosed by Dr. Fang Xiangling (Deputy Chief Physician, Child Health Department, Shenzhen Children's Hospital) according to DSM-5 criteria, will be enrolled after meeting inclusion/exclusion criteria. Participants will be recruited from the patient population of the Child Health Department and the International Medical Center at Shenzhen Children's Hospital.

#### 2.4.3 FMT fecal sample preparation

##### 2.4.3.1 Donor fecal collection

Fresh donor stool is collected and immediately transported in anaerobic bags to the FMT processing laboratory.

##### 2.4.3.2 Standardized FMT preparation protocol

###### 2.4.3.2.1 Facility

FMT preparation is conducted in a dedicated laboratory at Shenzhen Children's Hospital.

###### 2.4.3.2.2 Protocol

1. Reagents and Consumables:
  • 2.0 mL threaded centrifuge tubes (Axygen: MCT-200-C), 15 mL centrifuge tubes (Corning: 430791).
  • 150 mL sterile FMT storage bottles (SPL: 56125).
  • 0.9% saline (degassed), 80% glycerol (degassed).
  • Disposable sterile homogenization bags, 25 mL pipettes, 50 mL centrifuge tubes, sterile tongue depressors, spreaders.
  • GAM agar plates, reagent racks.
  • Ice packs, anaerobic containers, cryostorage boxes.

*Note*: All consumables must be sterile; alternative brands are permitted if specified items are unavailable.

**Figure 2:**
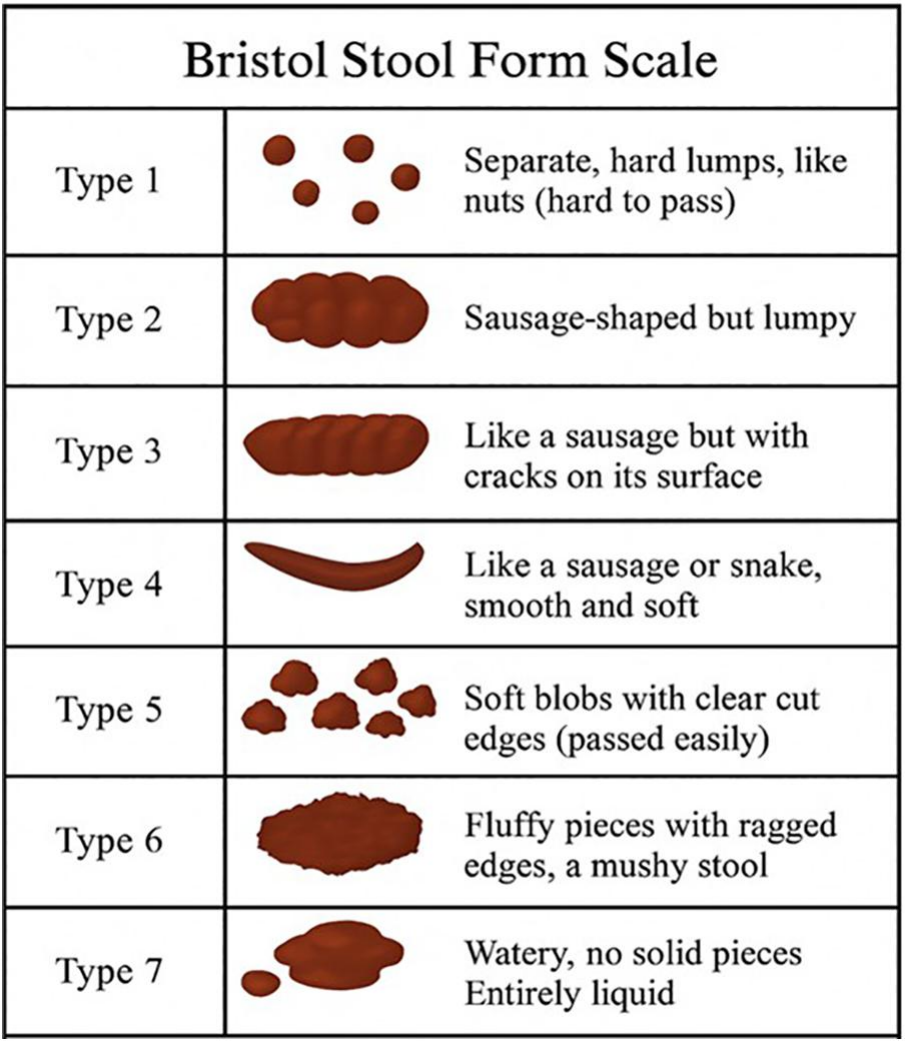
Bristol stool form scale. The figure illustrates the seven types of stool consistency. Adapted from Lewis and Heaton (56).

2. Equipment:
  Biological safety cabinet.
  Electronic balance.
  Stomacher homogenizer.
  −80 °C freezer.
  Pipettes.
3. Microbial Suspension Preparation:
  **Step 1**: Fresh stool is visually assessed using the Figure 2 Bristol Stool Form Scale (BSFS, type 3–5 are acceptable; type 1, 2, 6, or 7 result in sample rejection). If transportation compromises visual assessment, donor-provided Bristol scores are used.
  **Step 2**: Stool is weighed, mixed with 0.9% saline at a 1:3 ratio (e.g., 100 g stool + 300 mL saline), and homogenized for 5 min. Adjustments are made for samples <100 g.
  **Step 3**: The homogenate is filtered through a membrane-equipped bag using a stomacher (2 min).
  **Step 4**: Filtrate is transferred to sterile bottles, and glycerol is added to a final concentration of 10% (e.g., 87.5 mL filtrate + 12.5 mL 80% glycerol per 100 mL).
  **Step 5**: Bottles are labeled with donor ID and preparation time.
  **Step 6**: Suspensions are stored at −80 °C (stable for ≤6 months) or −20 °C (use within 1–4 weeks). Thaw at room temperature (<6 h) or in a water bath (≤37 °C) before administration.
  **Step 7**: A “Donor Sample Processing Record” is completed.
4. Residual Sample Handling:
  Remaining filtrate is aliquoted into 2 mL/15 mL centrifuge tubes with 10% glycerol and stored at −80 °C for quality control and post-treatment analysis.
  1 mL aliquots of saline and glycerol from the same batch are archived in 2.0 mL tubes.

###### 2.4.3.2.3 Sample Storage

All samples are stored long-term in −80 °C freezers at Shenzhen Children's Hospital.

##### 2.4.3.3 Quality control criteria

1. **Routine Stool Examination**: Random samples are tested for pathogens and cell counts.
2. **Pathogen Detection**: Negative results required for bacterial, parasitic, and viral pathogens; multidrug-resistant genes (e.g., ESBL, carbapenemase) must be absent.
3. **Viability Standards**: ≥80% bacterial viability; ≥5 × 10^8^ viable cells/mL.
4. **Microbial Consistency**:
  Uniform microbial composition within the same donor and batch.
  No statistically significant differences in microbiota between batches from the same donor within 6 months.
5. **Labeling**: Each bottle must be independently packaged and labeled.

#### 2.4.4 FMT administration protocol

##### 2.4.4.1 Pre-FMT preparation

Patients will begin a semi-liquid diet one day prior to the first FMT administration. Prior to the FMT procedure, all participants will undergo bowel preparation with polyethylene glycol (PEG) lavage to cleanse the intestinal tract, which is consistent with our clinical practice. Given that the FMT is delivered directly to the jejunum, the use of proton-pump inhibitors (PPIs) or other acid suppressants is not planned for this protocol.

##### 2.4.4.2 Nasojejunal tube placement, sedation, and risk management

Administration of FMT via the upper GI tract carries specific procedural considerations and risks that must be carefully managed in a pediatric population.

Procedure: The nasojejunal tube will be placed by an experienced pediatric gastroenterologist under the direct visualization of a video endoscope, confirming that the distal tip is correctly positioned in the jejunum past the ligament of Treitz.
Sedation: Recognizing that NJ tube placement can cause significant discomfort, anxiety, and requires patient cooperation, the procedure will be performed with appropriate sedation. Each child will undergo a pre-procedural assessment by a pediatric anesthesiologist to determine the optimal, individualized sedation plan. This plan may range from moderate (“conscious”) sedation to general anesthesia, depending on the child's age, anxiety level, and clinical condition, in accordance with the American Academy of Pediatrics guidelines for procedural sedation.
Risk Disclosure and Management: The informed consent process will explicitly detail the specific risks associated with upper GI tract administration and NJ tube placement. These risks include, but are not limited to: discomfort or pain during tube placement, risk of aspiration of gastric or fecal contents, potential for mucosal injury or nosebleed, tube dislodgement (e.g., due to coughing or vomiting), and the risks inherent to the sedation or anesthesia itself. Patients will be monitored closely by trained clinical staff during and after the procedure for any adverse events.

##### 2.4.4.3 FMT infusion and schedule

Once the NJ tube is confirmed to be in the correct position, patients will be positioned supine. The thawed fecal suspension (dose: 5 mL/kg) will be infused through the tube, followed by a 20 mL saline flush to clear the tube. After the infusion, patients will remain in a supine position for at least 30 min and will be required to fast for 2 h. This procedure will be repeated three times over a 5-day period (i.e., on Day 1, Day 3, and Day 5).

#### 2.4.5 Control of confounding variables

To minimize known confounding factors that can influence the gut microbiota, legal guardians will be instructed to maintain the child's typical diet and to avoid introducing major dietary changes (e.g., starting a gluten-free/casein-free diet) during the study period. A baseline 3-day dietary log will be collected. All concomitant medications and the use of supplements will be recorded at each study visit. While household environment cannot be controlled, data on significant dietary patterns and concomitant medications will be collected and considered as potential covariates in exploratory statistical models.

### 2.5 Outcome measures

The schedule for all assessments is detailed in Table 1.

**Table 1:** Visit schedule.

Time Window	Within 1 month pre-FMT (Baseline)	FMT treatment period*	1 week post-FMT	2 weeks post-FMT	3 weeks post-FMT	4 weeks post-FMT (±3 days)	5 weeks post-FMT (±3 days)	6 weeks post-FMT (±3 days)***	3 months post-FMT (±3 days)	6 months post-FMT (±3 days)	9 months post-FMT (±3 days)	12 months post-FMT (±3 days)***
Informed Consent	√											
General Data Collection	√	√										
Medical History Collection	√											
Vital Signs, Height, Weight	√	√	√	√	√	√	√	√	√	√	√	√
Inclusion/Exclusion Criteria	√											
Lab Tests**	√	√						√				√
Adverse Event Documentation		√	√	√	√	√	√	√	√	√	√	√
GSRS, DSR	√	√	√	√	√	√	√	√	√	√	√	√
CARS, SRS, ABC	√								√	√	√	√
Recipient Fecal Collection	√	√	√	√		√			√	√	√	√
Donor Fecal Collection	√											

* FMT treatment period: Includes 3 FMT administrations on Day 1, Day 3, and Day 5.

** Lab tests: Baseline tests include routine blood, urine, and stool tests, liver function (ALT, AST), renal function (BUN, Creatinine), coagulation profile, and ECG. Follow-up labs at 6 and 12 months will monitor long-term safety, including: (1) Immune/Inflammatory parameters (CBC with differential, hs-CRP, ESR) and (2) Metabolic parameters (Fasting Glucose, HbA1c, Fasting Lipid Profile, Liver Function Panel, Renal Function Panel).

*** Long-term safety & efficacy follow-ups will specifically monitor long-term safety, including immune, metabolic, and neurodevelopmental parameters.

#### 2.5.1 Primary outcomes

1. **GSRS.**

a. **Timeframe**: Pre-FMT, during FMT, and at 1, 2, 3, 4, 5, 6 weeks, 3 months, 6 months,9 months and 12months post-FMT.
b. **Description**: A 15-item questionnaire evaluating GI symptom severity. Each item is scored from 1 (“none”) to 7 (“very severe”), with descriptors: 1 (none), 2 (mild), 3 (slight), 4 (moderate), 5 (marked), 6 (severe), 7 (very severe). Participants with <50% reduction in mean GSRS scores are classified as non-responders.

**2. CARS**

a. **Timeframe**: Pre-FMT, 3 months, 6 months,9 months and 12 months post-FMT.
b. **Description**: Administered by trained assessors, this 15-item scale yields a total score ranging from 15 to 60. Scores are interpreted as: <30 (non-autistic), 30–36 (mild-to-moderate autism), and ≥36 (moderate-to-severe autism).

#### 2.5.2 Secondary outcomes

1. Daily Stool Record (DSR)
  a. **Timeframe**: Pre-FMT, during FMT, and at 1, 2, 3, 4, 5, 6 weeks, 3 months, 6 months, 9 months and 12 months post-FMT.
  b. **Description**: Daily documentation of stool characteristics using the Bristol Stool Scale. The percentage of days with abnormal bowel movements (e.g., abnormal consistency, absence of stool, or use of GI medications) is calculated, with higher percentages indicating worse symptoms.
2. Social Responsiveness Scale (SRS)
  a. **Timeframe**: Pre-FMT, 3 months, 6 months,9 months and 12 months post-FMT.
  b. **Description**: A 65-item questionnaire assessing social skills. Items are scored 0 (“never true”) to 3 (“almost always true”), with total scores ranging from 0 to 195. Higher scores indicate greater social impairment.
3. Aberrant Behavior Checklist (ABC)
  a. **Timeframe**: Pre-FMT, 3 months, 6 months,9 months and 12 months post-FMT.
  b. **Description**: A 58-item checklist evaluating maladaptive behaviors. Total scores range from 0 to 174, with ≥31 indicating autism tendency and >53 confirming an autism diagnosis. Higher scores reflect more severe behavioral issues.
4. Microbiota Analysis
  a. **Sequencing Method:** Fecal samples will be subjected to metagenomic next-generation sequencing (mNGS) to comprehensively characterize the gut microbiota at both taxonomic and functional levels. Total microbial DNA will be extracted from fecal samples, followed by library construction and shotgun sequencing on an Illumina NovaSeq platform (or equivalent), aiming for a sequencing depth of at least 10 Gbp per sample to ensure adequate coverage for both abundant and rare species, as well as functional gene analysis.
  b. Bioinformatic and Statistical Analysis Pipeline:

The raw sequencing data and subsequent analyses will adhere to the following rigorous bioinformatics procedure to ensure accuracy and reproducibility.

Step 1: Raw Data Quality Control.

To assess sequencing quality and detect potential contamination, FastQC (Version 0.12.1) (36) and MultiQC (Version 1.15) (37) will be utilized with default parameters. Key quality metrics include ≥80% of bases with a Q-score >30, adapter content <5%, and GC distribution within the expected range of 40%–50%. The output report, ASD_FMT_Raw_QC.html, will be reviewed to exclude samples that fail to meet these criteria.

Step 2: Read Trimming and Host DNA Removal.

In this step, KneadData (Version 0.12.0) (38) will be used to perform read trimming and host DNA removal. Using the GRCh38.p14 human reference genome, KneadData will first trim the reads to remove low-quality bases and adapters, then filter out any reads mapping to the human genome. This will ensure that host-derived contamination is minimized to ≤5%. After the filtering process, the remaining high-quality clean reads are expected to constitute at least 70% of the original raw reads, providing an enriched dataset of non-host sequences for subsequent analyses.

STEP 3: Taxonomic Profiling.

After obtaining the high-quality clean reads, high-specificity taxonomic profiling will be performed to classify the microbial community at the species level. This will be achieved using MetaPhlAn4 (Version 4.1.1) (39) and the database mpa_vJun23_CHOCOPhlAnSGB_202403, which maps the sequencing reads to a set of species-specific marker genes. Beside the default parameters, the option “-s” will be added in the parameter so as to generate SAM files for raw sequence alignment output. The result will be a detailed table showing the relative abundance of each species present in the samples, enabling precise classification and valuable insights into the microbial community composition of the samples.

STEP 4: Functional Profiling.

Following taxonomic profiling, functional profiling will be performed to assess the functional capabilities of the microbial communities. This will be conducted using HUMAnN3 (Version 3.8) (40), which maps the high-quality, host-depleted reads to known gene families and metabolic pathways, offering a detailed functional profile of the microbial communities. The output will include a table of relative abundances of predicted pathways and gene families, with particular attention to those pathways that may be relevant to the effects of FMT. This will help identify functional shifts in microbial communities post-FMT, offering insights into the metabolic mechanisms potentially linked to improvements in ASD symptoms.

STEP 5: *de novo* Assembly and MAG Reconstruction.

For *de novo* assembly of the metagenomic data, Megahit (Version 1.2.9) (41) will be used. This step involves assembling the high-quality, host-depleted reads into longer contiguous sequences (contigs) for further analyses. The assembly will be performed with the following parameters: –k-list 21,29,39,59,79,99,119,141\–min-contig-len 1,000\–presets meta-large\–memory 0.95\–cpu-only. After that, metagenomic binning and bin refinement will be conducted using MetaWRAP (42).The MetaWRAP refinement aims to enhance the quality of our MAG (metagenome-assembled genomes) binning, utilizing the parameters -c 50 -x 10, which retains only bins with completeness greater than 50% and contamination less than 10%. Redundancies in the MAGs will be removed with dRep Version 2.6.2 (43). MAGs will be annotated using GTDBtk Version 2.3.2 (44), and their quality will be evaluated using CheckM Version 1.0.7 (45). Gene prediction will be performed using Prodigal Version 2.6.3 (46), clustering of genes with CD-HIT Version 4.8.1 (47).

### 2.6 Safety assessment

Safety monitoring will include documentation of adverse events (AEs), serious adverse events (SAEs), and abnormal laboratory parameters throughout the study, extending to the 12-month final follow-up.

1. Definitions and Grading: AEs will be defined as any untoward medical occurrence in a participant, regardless of its suspected relationship to the intervention. SAEs will be defined as any AE that results in death, is life-threatening, requires inpatient hospitalization, or results in persistent disability. All AEs will be graded for severity using the Common Terminology Criteria for Adverse Events (CTCAE v5.0, 2018).
2. Causality Assessment: The Principal Investigator (PI) will assess all AEs for their potential relationship to the FMT intervention (classified as unrelated, unlikely, possible, probable, or definitely related).
3. SAE Reporting: All SAEs will be reported by the study team to the PI within 24 h of awareness. The PI will then report the SAE to the Shenzhen Children's Hospital Ethics Committee (EC) within 7 days.
4. Long-Term Safety Monitoring: (New section for comment 6) In addition to routine AE/SAE reporting, the extended 12-month follow-up will specifically monitor for potential long-term risks. This monitoring will include:
  a. Neurodevelopmental Parameters: Assessment for any new onset or significant worsening of neurological or psychiatric symptoms using the standardized scales (CARS, SRS, ABC) and clinical evaluation.
  b. Immune and Inflammatory Parameters: Monitoring for new onset of allergic, atopic, or autoimmune symptoms. Laboratory tests at 6 and 12 months will include Complete Blood Count (CBC) with differential, high-sensitivity C-reactive protein (hs-CRP), and Erythrocyte Sedimentation Rate (ESR).
  c. Metabolic Parameters: Laboratory tests at 6 and 12 months will include Fasting Blood Glucose, Glycated Hemoglobin (HbA1c), a Fasting Lipid Profile, a Liver Function Panel (e.g., ALT, AST, ALP, Total Bilirubin), and a Renal Function Panel (e.g., BUN, Creatinine) to monitor for metabolic disturbances.
  d. GI Status: Continued monitoring of GI symptoms via GSRS and DSR.
5. Data and Safety Monitoring Board (DSMB): Given the interventional nature of the study and the vulnerable pediatric population, an independent DSMB will be established. The DSMB will be composed of at least three independent experts (e.g., a pediatric gastroenterologist, a child psychiatrist, and a biostatistician) not affiliated with the study. The DSMB will convene to review cumulative safety data after the first 10 participants have completed the 6-week follow-up, and thereafter as needed, to protect participant safety and ensure study integrity.

### 2.7 Statistical analysis

#### 2.7.1 Analysis of clinical outcomes

Analysis of clinical data (CARS, GSRS, SRS, ABC) will be conducted using SPSS 26.0. Continuous variables will be expressed as mean ± standard deviation. For comparisons between baseline and specific follow-up points, paired *t*-tests or Wilcoxon signed-rank tests will be used, as appropriate based on data distribution. To analyze the longitudinal changes in primary and secondary outcomes over the multiple time points, linear mixed-effects models will be employed to account for intra-subject correlations.

To ensure the highest standard of statistical analysis, we have formally engaged a qualified statistician as a consultant for this study. The statistician will be involved in the final data analysis plan, execution, and interpretation of results.

The primary analysis of clinical outcomes will be conducted on the per-protocol (PP) population, defined as all participants who complete the primary endpoint assessment at 12 months. A secondary analysis will be performed on the intent-to-treat (ITT) population, which includes all enrolled participants who have received at least one Fecal Microbiota Transplantation administration. For longitudinal data (e.g., GSRS), linear mixed-effects models are inherently robust to data missing at random. For missing primary endpoint data (e.g., 12-month CARS), multiple imputation techniques will be explored and applied if necessary, under the guidance of our study statistician.

#### 2.7.2 Analysis of microbiota data

All microbiota-related statistical analyses will be conducted within R 4.5.1 (48).

Diversity Analysis: To calculate for both taxonomic and functional profiles to assess changes in community richness, evenness, and structure across different time points of FMT, diversity analysis will be conducted with the R package vegan version 2.7–2 (49) Alpha diversity (within-sample diversity, such as the Shannon index and Simpson index) and beta diversity (between-sample diversity, e.g., Bray-Curtis dissimilarity) will be calculated.

Differential Abundance Analysis: To identify taxa and functional pathways that exhibit significant changes, linear discriminate analysis with effect size (LEfSe) analysis (50) will be used with microeco package Version 1.15. 0 (51). After that, to identify associations between microbiota profiling and clinical improvements in core ASD symptoms or GI comorbidities (scores of ABC, CARS, SRS and GSRS), multivariate association with linear models (MaAsLin) analysis will be performed by Maaslin2 package Version 1.22.0 (52), followed by Benjamini-Hochberg correction for multiple hypothesis tests (53).

Statistical analysis (Microbiota): To determine differential abundance of metagenomic features, normality and homogeneity tests on all data will be conducted firstly. Measurement data such will be expressed as mean ± SE for normally distributed data and as median (P25, P75) for nonnormally distributed data. For normally distributed data with homogeneous variances in 2-group comparisons, paired *t*-tests will be used. Nonnormally distributed or nonhomogeneous data will be analyzed using nonparametric Wilcoxon tests. In multiple group comparisons, normally distributed data with homogeneous variances will be analyzed with analysis of variance, while nonnormally distributed or nonhomogeneous data will be analyzed with nonparametric Kruskal–Wallis tests (54). Correlations will be calculated between clinical and metagenomic features with Spearman's correlation. *P*-values will be adjusted with false discovery rate (FDR) with the method from Benjamini-Hochberg (53) when multiple hypotheses are considered simultaneously and will be denoted adj. P. All graphs will be visualized using the ggplot2 package (Version 4.0.0) (55).

### 2.8 Sample size calculation

The sample size for this prospective single-arm study was determined based on the primary outcome of the change in the CARS score from baseline to 12 months post-intervention. The calculation is based on the 12-month follow-up data from a large-scale preliminary study by Ye et al. (2022) on FMT for children with ASD (31).

In that study, the mean CARS score in children with ASD before treatment was 35.2 ± 6.5. Following FMT, the mean score at 12 months post-treatment was 31.0 ± 6.7. This represents a mean reduction (δ) of 4.2 points, which is considered the clinically significant difference to be detected in this protocol.

The standard deviation of the difference (*σd*) is required for the calculation. Assuming a conservative correlation coefficient (*r*) of 0.5 between baseline and 6-month measurements (a common assumption for pre-post study designs), the standard deviation of the difference can be estimated using the formula:

$$\sigma_{d}=\sqrt{\sigma_{\;pre}^{2}+\sigma_{\;post}^{2}-2r\sigma_{\;pre}\sigma_{\;post}}$$

This yields an estimated *σd* of approximately 6.60.

Using a two-sided paired *t*-test, with a significance level (*α*) of 0.05 and a desired statistical power (1-*β*) of 80% $\left(Z_{\beta }=0.8416\right)$, the required sample size (*n*) was calculated using the following formula: $n=(Z_{\alpha /2}+Z_{\beta }{)}^{2}\sigma_{d}^{2}/\delta^{2}$.Therefore, *n* = [(1.96 + 0.8416)^2^ × 6.60^2^]/(4.2^2^) ≈ 19.4.

Based on these parameters, the minimum required sample size is calculated to be 20 participants.

To account for a potential dropout rate of approximately 20% during the 6-month follow-up period, the sample size needs to be adjusted.

Adjusted sample size = *n*/(1—dropout rate) = 20/(1–0.20) = 25.

Therefore, the planned enrollment of 30 participants is more than sufficientto provide adequate statistical power for evaluating the primary outcome of this study.

## 3 Discussion

ASD constitutes a complex neurodevelopmental disorder with an escalating global prevalence, representing a critical public health challenge. Up to 90% of children with ASD exhibit comorbid GI symptoms which correlate with ASD severity and may exacerbate symptoms through bidirectional interactions with gut microbial dysbiosis.

The MGBA has emerged as a pivotal focus in ASD pathogenesis. Evidence confirms substantial intestinal dysbiosis in ASD populations. FMT is an innovative therapeutic strategy that offers novel potential for ASD intervention by reconstructing the gut ecology. Preliminary studies demonstrate FMT's capacity to reduce ASD and GI symptom scores and sustain improvements in microbial diversity.

However, critical translational barriers persist, including inconsistent efficacy, safety uncertainties, and unclear mechanisms. This prospective single-center single-arm interventional study addresses these gaps by evaluating multi-course FMT efficacy and safety in moderate-to-severe ASD children.

The study has some limitations. As a single-arm, open-label trial, this study cannot completely exclude the potential influences of placebo effects, natural disease progression, or observer bias. This design was chosen with reference to previous foundational studies in the field, such as Kang et al. (29), which utilized a similar open-label approach to provide the first critical evidence of FMT's efficacy. This study is intended as an exploratory trial to provide preliminary evidence and valuable data from the Chinese population, which will be invaluable for designing future large-scale, placebo-controlled trials. The absence of healthy controls also limits clinical benchmarking. Furthermore, the use of a single donor, while a deliberate choice to minimize inter-donor variability as a confounding factor in this preliminary efficacy study, may limit the generalizability of the findings, as the concept of an “ideal donor” is still under investigation.

To counterbalance these limitations, the protocol incorporates rigorous safety oversight via an independent DSMB and extends follow-up to 12 months to assess long-term outcomes. Additionally, the employment of deep mNGS will provide high-resolution insights into microbial engraftment and functional pathways.

Ultimately, the data generated from this study will provide crucial preliminary evidence and effect size estimates essential for designing future large-scale, randomized placebo-controlled trials, helping to catalyze the translation of microbiota-targeted therapies from bench to pediatric clinical practice.
